# PI3K/Akt/mTOR, a Pathway Less Recognized for Staphylococcal Superantigen-Induced Toxicity

**DOI:** 10.3390/toxins4111343

**Published:** 2012-11-15

**Authors:** Teresa Krakauer

**Affiliations:** Department of Immunology, Integrated Toxicology Division, United States Army Medical Research Institute of Infectious Diseases, Fort Detrick, Frederick, MD 21702, USA; Email: teresa.krakauer@us.army.mil; Tel.: +1-301-619-4733; Fax: +1-301-619-2348

**Keywords:** staphylococcal superantigens, inflammatory cytokines, signaling pathways, PI3K, Akt, mTOR, therapeutics

## Abstract

Immunostimulating staphylococcal enterotoxin B (SEB) and related superantigenic toxins cause diseases in humans and laboratory animals by activating cells of the immune system. These toxins bind directly to the major histocompatibility complex (MHC) class II molecules on antigen-presenting cells and specific Vβ regions of T-cell receptors (TCR), resulting in hyperactivation of both T lymphocytes and monocytes/macrophages. Activated host cells produce excessive amounts of proinflammatory cytokines and chemokines, especially tumor necrosis factor α, interleukin 1 (IL-1), IL-2, interferon γ (IFNγ), and macrophage chemoattractant protein 1 causing clinical symptoms of fever, hypotension, and shock. The well-explored signal transduction pathways for SEB-induced toxicity downstream from TCR/MHC ligation and interaction of cell surface co-stimulatory molecules include the mitogen-activated protein kinase cascades and cytokine receptor signaling, culminating in NFκB activation. Independently, IL-2, IFNγ, and chemokines from activated T cells signal via the phosphoinositide 3-kinase (PI3K), the serine/threonine kinases, Akt and mammalian target of rapamycin (mTOR) pathways. This article reviews the signaling molecules induced by superantigens in the activation of PI3K/Akt/mTOR pathways leading to staphylococcal superantigen-induced toxicity and updates potential therapeutics against superantigens.

## 1. Introduction

*Staphylococcus aureus* is a ubiquitous gram-positive coccus that produces several exotoxins with potent immunostimulating activities which contribute to its ability to cause disease in humans, most notably food poisoning, toxic shock, and autoimmune diseases [1,2,3,4,5,6,7]. Staphylococcal enterotoxins A through U (SEA-SEU) and toxic shock syndrome toxin 1 (TSST-1) were termed “superantigens” due to their ability to polyclonally activate T cells at picomolar concentrations. Since then, many structurally similar superantigens from *Staphylococcus aureus* and *Streptococcus pyrogenes,* as well as those from other bacteria, virus, and fungal origins have been discovered [7]. Staphylococcal superantigens induce a mitogenic response in T cells, stimulating a large proportion (5%–30%) of T cells to proliferate compared to less than 0.01% of T-cell proliferation initiated by a conventional antigen [8]. Superantigen binds outside the peptide-binding groove of the major histocompatibility complex (MHC) class II and bypasses conventional antigen processing by antigen-presenting cells (APC) [3,7,8]. By interacting with both MHC class II molecules on APC and specific elements within the variable region of the Vβ chains of the T cell receptor (TCR), these microbial toxins perturb the immune system and induce high levels of proinflammatory cytokines and chemokines [9,10,11,12,13,14,15,16]. Other tissue damaging molecules such as matrix metalloproteinases (MMPs) and tissue factor are also produced by superantigen-activated host cells, affecting both inflammatory and coagulation pathways [17]. Activated neutrophils produce reactive oxygen species (ROS) which leads to increased vascular permeability and lung injury [18]. Tumor necrosis factor α (TNFα) and interleukin 1 (IL-1) are induced early after intoxication and are direct mediators of fever, hypotension, and shock [19,20,21]. In addition, IFNγ produced by activated T cells acts synergistically with TNFα and IL-1 to enhance host defense and tissue injury by establishing an inflammatory environment for T cell activation and differentiation. IL-2, another cytokine from superantigen-activated T cells is essential for T-cell growth but excessive amounts cause vasodilation leading to vascular leak and edema [22].

SEB has historically been the most intensively studied superantigen and is listed as a category B select agent by the Centers for Disease Control and Prevention (CDC), as it can be used as an air-borne, food-borne, and water-borne toxin. Depending on the dose and route of exposure, SEB and other SEs cause food poisoning, acute and fatal respiratory distress, autoimmune diseases, and toxic shock [3,23,24,25,26,27]. Superantigens also enhance proinflammatory response and lethality by synergizing with other bacterial products such as lipopolysaccharide (LPS), lipoproteins, and viruses [28,29,30,31]. Recent studies further indicate that superantigens upregulate toll-like receptor 2 (TLR2) and TLR4, receptors for binding pathogen associated molecular patterns, further amplifying the immune response to other microbial products [32,33]. Because it is common to encounter pathogens and their toxins concomitantly in real life, superantigens can have profound toxic effects at extremely low concentrations.

## 2. Staphylococcal Superantigen Structure and Binding

Staphylococcal enterotoxins (SEs) and TSST-1 are 22-kD to 30-kD single-chain proteins with well-characterized secondary and tertiary structures [34]. Staphylococcal superantigens are grouped based on their primary sequence homology with SEA, SED, and SEE as the first group sharing the highest sequence homology of 53% to 81% [5,7,35]. A second group consists of SEB, the SECs, and SEG, which are 50% to 66% homologous. TSST-1 stands alone by itself in one group as it is distantly related, with only 28% homology and has a distinct, shorter primary sequence of 194 amino acids with no cysteines and a missing “disulfide loop” commonly found in SEs. A study with mutants of SEC2 indicated that the disulfide loop may be responsible for the emetic activity of SEs [36]. A newer classification scheme of five bacterial superantigen groups including the streptococcal superantigens was proposed based on their phylogenic relationships and similarities in modes of binding to MHC class II molecules. Cross-reactivities of polyclonal and monoclonal antibodies to the SEs and TSST-1 indicate common epitopes exist among these toxins [37]. X-ray crystallography of SEA, SEB and TSST-1 reveals similarities in the secondary-tertiary structure with two tightly packed domains containing β-sheets and α-helices [34]. The relatively conserved TCR-binding site is located in the shallow groove between these two domains [7,34,38,39]. 

There are two distinct sites on MHC class II molecules for superantigen binding; a common, low-affinity binding site located on the α-chain of MHC class II and a high-affinity, zinc-dependent binding site on the β-chain [7,40,41,42,43]. Superantigens in the SEA subfamily bind to both sites, whereas SEB and TSST-1 bind only to the generic low-affinity site [41,42,43,44,45]. Individual toxin displays preferential binding to distinct alleles of specific MHC isotypes accounting for differences in host responses to SEs [45,46,47,48]. In general, HLA-DR binds SEs and TSST-1 better than HLA-DP or -DQ, and murine IE molecules bind with higher affinity than IA [45,48].

The binding of superantigens to TCR Vβ is of low affinity (*K*_d _= 10^−4^–10^−6^ M), similar to those with conventional MHC/peptide/TCR [49,50]. However, each toxin binds to a distinct repertoire of TCR Vβ chains revealing unique Vβ specificities of individual superantigen [7,51]. The binding contacts are mostly between the side-chain atoms of the superantigen and the complementarity-determining regions 1 and 2 and the hypervariable region 4 within the Vβ chain. There are multiple modes of superantigen binding to MHC and TCR. SEB and SEC crosslink MHC class II α chain and TCR Vβ whereas SEA binds to both α and β chain of MHC class II to crosslink TCR Vβ [7]. The cooperative binding of superantigen/MHC complex with TCR enables superantigen binding to TCR with a higher affinity than with toxin alone [49]. A recent study suggests a third binding site for the co-stimulatory receptor CD28 on T cells to SEB and peptides derived from the CD28 binding region protected mice from SEB-induced lethality [52]. Receptor clustering and subsequent intracellular signaling in both T cells and APC lead to excessive mediator release and specific pathways of cell activation [53,54].

**Figure 1:**
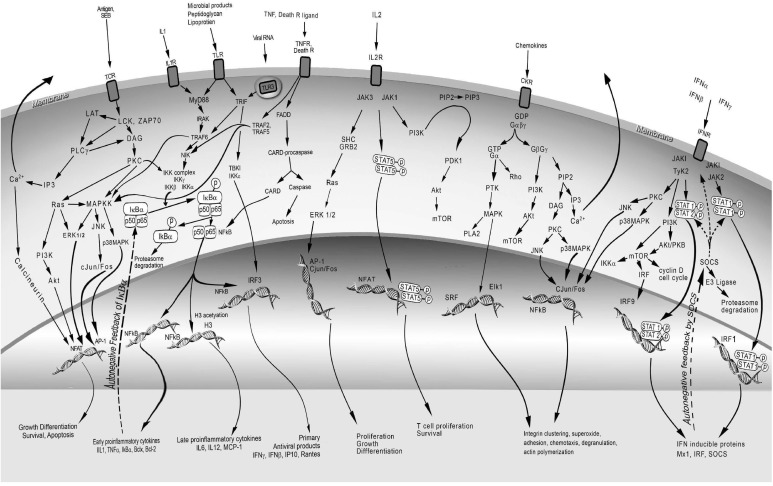
Cell receptors, intracellular signaling molecules, and signal transduction pathways used by superantigens and mediators induced by superantigens.

## 3. The Three Signals for T Cell Activation

Superantigens interact with both CD4^+^ and CD8^+^ T cells as well as mononuclear phagocytes bearing MHC class II molecules [55,56,57,58]. Interaction of superantigen with TCR provides signal 1 for T cell activation. As with conventional antigens, signal 2 comes from the engagement of co-stimulatory molecules on APC and T cells upon superantigen binding and optimizes T cell activation [59,60]. Expression of intercellular adhesion molecule (ICAM) on APC promotes stable cell conjugate formation and allows immunological synapse to occur. Initiation of TCR signaling by the formation of supramolecular activation clusters comprising of TCR, co-stimulatory molecule CD28 and signaling kinases is F-actin dependent [61]. The interactions of adhesion molecules and co-stimulatory molecules, LFA-1/ICAM-1 and CD28/CD80, have both been implicated in SEA- and SEB-mediated T-cell activation [60,61,62]. Activation of the CD28-regulated signal transduction pathway during SEA stimulation of T cells enhanced IL-2 mRNA stability [62]. CD28 co-stimulation also increases T cell survival by enhancing the expression of Bcl-xl [63]. Blocking CD28 with short synthetic peptides corresponding to the binding region of CD28 inhibited TNFα, IFNγ, and IL-2 [52]. Other cell surface molecules such as CD2, CD11a/ICAM-1, and ELAM facilitated optimal activation of endothelial cells and T cells by SEB [60]. TCR and costimulatory receptors activate signaling kinases, protein kinase C (PKC) and protein tyrosine kinases (PTKs) by the release of intracellular second messengers and various intracellular adaptors [64,65,66]. PKC and PTK activation lead to other downstream signaling pathways including mitogen–activated protein kinase (MAPK), extracellular signal regulated kinase (ERK) and c-jun *N*-terminal kinase (JNK) pathways ultimately activating transcriptional factors NFκB, NF-AT, and AP-1 [65,66,67]. Many proinflammatory cytokine genes contain NFκB binding sites in the promotor/enhancer region and are induced by NFκB [68]. The cytokines IL-1, TNFα, IFNγ, IL-2, IL-6, and chemokines, specifically MCP-1 are induced directly by superantigens, representing the third signal for T cell activation. IL-1 and TNFα can also activate fibroblasts, epithelial, and endothelial cells to produce other mediators providing inflammatory stimuli for activation of many different cell types [21]. The mediators produced by superantigen-activated cells exert profound effects on the immune and cardiovascular system, culminating in multi-organ dysfunction and lethal shock. PTKs and T cell cytokines also activate the lipid kinase, phosphoinositide 3 kinase (PI3K) affecting many intracellular processes including cell survival, growth, and migration [69]. PI3K consists of eight isoforms, regulates many physiological and pathological processes, and plays a key role in cancer, being constitutively active in malignancy and promotes growth factor independent growth in tumor cells. 

## 4. TCR and Costimulatory Receptors Activate the Phosphatidylinositol Pathway

T cell activation via the TCR-CD3 complex induces the activation of the Src family PTKs, LCK and FYN, which in turn phosphorylate tyrosine-based motifs of the TCR intracellular components and other cellular substrates [64,65,66]. LCK activates another PTK, ZAP-70, which then induces tyrosine phosphorylation of the adaptors LAT (linker for activation of T cells) and SLP-76 (SH2-domain-containing leukocyte protein-76). These adaptors help to localize phospholipase C γ (PLCγ) to the plasma membrane and activate PLCγ through phosphorylation by TCR-induced kinases, LCK and ZAP-70 (Figure 1) [64,65,66]. Phosphorylated and activated PLCγ cleaves phospholipid phosphatidylinositol 4,5-bisphosphate, generating the second messengers diacylglycerol (DAG) and inositol 1,4,5-trisphosphate (IP3). DAG activates protein kinase C θ (PKCθ) and indirectly the protooncogene Ras whereas IP3 binds to its receptor on the surface of the endoplasmic reticulum and induces an increase in intracellular calcium. PTKs also activate PI3K upon specific ligand binding to a number of receptors besides the TCR, including CD28, IL-2 receptor (IL-2R), insulin receptor, growth factor receptor, and G-protein-coupled receptor (GPCR). Activation of PI3K by PTK leads to the generation of several inositol phospholipids including phosphatidylinositol 3,4-bisphosphate (PIP2) and phosphatidylinositol 3,4,5-trisphosphate (PIP3) [64]. PIP3 recruits phosphoinositide-dependent kinase 1 (PDK1) to the plasma membrane and activates it by phosphorylation. Activated PDK1 then phosphorylates Akt and PKCθ [70]. Although the activation of PKCθ isoform in superantigen-activated cell has not been defined, PKCθ can be found at immunological synapse formed after T cell activation by anti-CD3 and anti-CD28 [71]. Activation of PKCθ leads to the phosphorylation of target genes, one of which is the activation of the inhibitor of κB (IκB) kinase complex (IKK) [70]. IKK phosphorylation of IκB leads to its degradation, releasing NF-κB to be translocated to the nucleus where it binds and activates many NFκB target genes. Another kinase which is inducible by high cellular AMP/ATP ratio called AMP-activated protein kinase (AMPK) can also phosphorylate PKCθ [72]. The multiple phosphorylation sites on PKCθ allow for its regulation by at least three different kinases, LCK, PDK1 and AMPK, coordinating input from external stimuli. 

The superantigen TSST-1 induces inositol phospholipid turnover, protein kinase C translocation, and calcium mobilization in human T cells resembling responses from those of a mitogenic signal [73]. Various PTK inhibitors were used to study the PTK and PI3K pathways in mediating the effects of superantigens. The production of IL-1 by TSST-1-stimulated human macrophage cell line was blocked by three PTK inhibitors, genistein, tyrphostin, and herbimycin A [74]. However these inhibitors are not very specific as genistein can also block the activity of PKA and PKC. The exact PTK or sites of inhibition have not been identified with newer antibodies available for each specific PTK. Other PI3K inhibitors, wortmannin and LY294004 have not been tested with superantigen-activated cells. *In vivo* studies using these inhibitors on superantigen-induced shock models are lacking, perhaps due to inherent toxicity, non-specificity, and the existence of different PI3K isoforms. Recently, the superantigen SEE was shown to use an alternative LCK-independent pathway by activating PLCβ signaling in T cells [75].

## 5. Regulation of Akt and Mammalian Target of Rapamycin (mTOR)

**Figure 2:**
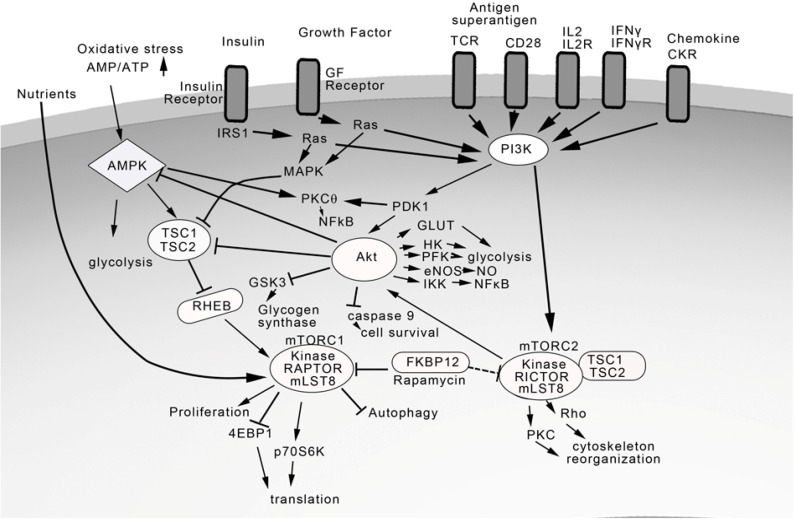
The PI3K/Akt/mTOR pathway in superantigen activation.

Downstream of PI3K is the serine/threonine kinase Akt which mediates many diverse biological processes such as glucose transport, glycolysis, glycogen synthesis, cell proliferation, NFκB activation, and inhibition of apoptosis [76,77] (Figure 2). Similar to PDK1, Akt can also be recruited to the plasma membrane by the lipid messenger PIP3. The activation of Akt is controlled by two main phosphorylation sites. Phosphorylation of the activation loop of Akt at Thr-308 by PDK1 is essential for activation whereas phosphorylation of Ser-473 within the regulatory region further enhances its activity. The role of Akt in SEB-mediated cellular effects has not been defined due to the lack of specific inhibitors, but its activation downstream of PI3K indicates the importance of Akt upon the binding of several specific ligands as diverse as antigens/superantigens, IL-2, insulin, growth factor, chemokines to their receptors TCR, IL-2R, insulin receptor, receptor tyrosine kinase (RTK), and GPCR, respectively. Two potent cytokines from superantigen-stimulated T cells, IFNγ and IL-2 also activate PI3K/Akt pathway via the transducer Janus kinase 1 (JAK1) after binding to the IFNγ and IL-2 receptor, respectively [78,79].

One of the downstream targets of Akt in controlling cell proliferation and protein translation is mTOR [80,81,82]. mTOR is a serine/threonine kinase that exists as two separate complexes, mTOR complex1 (mTORC1) and mTORC2 and they do not interact directly. mTORC1 comprises of a kinase component and two highly conserved proteins raptor and mLST8. A specific inhibitor, rapamycin, binds to the immunophilin FK506-binding protein 12 (FKBP12) which then blocks mTORC1 activity specifically [83]. Rapamycin has been used extensively to study the functions of mTORC1 and mTORC2 in cell activation [83]. The action of rapamycin on mTORC2 is controversial, with earlier reports of lack of inhibition to more recent studies indicating partial inhibition of mTORC2 with prolonged treatment with rapamycin [84]. The most important function of mTORC2 lies upstream since mTORC2 enhances Akt activity by phosphorylating Akt on Ser-473. 

A critical protein complex in the regulation of Akt/mTOR is the TSC1/TSC2 (tuberous sclerosis complex 1 and 2) which acts as a negative regulator of mTORC1 [80,81,82,83,84,85,86]. Phosphorylation of TSC2 by Akt results in the suppression of TSC1/TSC2 inactivation of the small GTPase, RHEB (Ras homologue enriched in brain). Because RHEB is a potent activator of mTORC1, the effect of Akt on TSC1/TSC2 is to promote mTORC1 activity. In contrast, TSC1/TSC2 associates with mTORC2 and promotes mTORC2 to phosphorylate and activate Akt. Cells deficient in TSC1/TSC2 complex are defective in both mTORC2 and Akt activity. Activation of mTORC1 leads to phosphorylation and activation of the ribosomal 40S protein p70S6 kinase (p70S6K) and the eukaryotic initiation factor binding protein 1 (4EBP1) [80,81,82,83]. Phosphorylated p70S6K promotes mRNA translation and cell growth by enhancing the biosynthesis machinery inside the cell. The phosphorylation of 4EBP1 prevents it from inhibiting the initiation factor EIF4E, thereby stimulating protein synthesis. Rapamycin blocks mTORC1 and inhibits the translation of proteins that are essential to G1 to S phase transition. mTORC2 can be stimulated by growth factors directly via PI3K promoting downstream PKC activity through phosphorylation and activating Rho, culminating in actin reorganization. mTORC1 and mTORC2 have distinct activities and act to coordinate signaling pathways from mitogenic and superantigenic signals, growth factors and cytokines via PI3K/Akt. mTORC1 can also be stimulated directly by nutrients such as amino acids and inactivated by oxidative stress or low cellular energy levels via AMPK which activates TSC1/TSC2 by phosphorylation leading to mTORC1 inhibition [85,86]. Adding another level of regulation, Akt can inhibit AMPK through phosphorylation and activates mTORC1. The cross-regulation of the components of the PI3K/Akt/mTOR pathway allows for the tight control on energy levels, metabolism, proliferation, and growth. 

Many excellent reviews have been written on the PI3K/Akt/mTOR pathway with original reference citations to novel observations and the details of signaling molecules [80,81,82,83,84,85,86]. A critical role for mTORC in SEB-induced signaling events is evident from the efficacy of the mTORC1-specific inhibitor, rapamycin in rescuing mice from SEB-induced shock [87]. Rapamycin inhibited SEB-induced T cell proliferation and was also a potent inhibitor of SEB-induced IL-2 and IFNγ *in vitro* and *in vivo*. Furthermore, in comparison with dexamethasone treatment in the same mouse model of SEB-mediated shock, early administration of dexamethasone post-SEB exposure as well as longer duration of treatment was necessary to prevent lethality. The SEB-induced PI3K/Akt/mTORC axis is found to be more effectively inhibited even when rapamycin was applied at a later time (24 h) after SEB exposure with shorter duration of treatment sufficient to block SEB-induced shock [87]. 

## 6. Proinflammatory Mediators Signal via NFκB Activation

Excessive release of proinflammatory cytokines mediates the toxic effects of superantigens. The proinflammatory cytokines IL-1 and TNFα can directly activate the transcriptional factor NFκB in many cell types that include epithelial and endothelial cells thus perpetuating the inflammatory response. The receptors, adaptors, and the signaling molecules used by IL-1, TNFα, IL-2, IL-6, and IFNγ are vastly different and represent five different families of cytokine receptors. 

IL-1 interacts with IL-1 receptor 1 (IL-1R1) requiring an additional receptor accessory protein for subsequent activation of downstream signaling molecules, the adaptor myeloid differentiation factor 88 (MyD88), IL-1R-associated protein kinase 1 (IRAK1), and TNF receptor-associated factor 6 (TRAF-6) [88]. Another set of related receptors, the toll-like receptors (TLRs), signal with similar intracellular adaptors and molecules as those used for IL-1R1 (Figure 1) but are not used for superantigen signaling. However, SEB was reported to increase cellular expression of TLR2 and TLR4 [32,33]. The TLRs are conserved type 1 transmembrane receptors used by pathogen associated molecules to stimulate host innate immune responses and influence the adaptive immune response [89]. There is some specificity of individual TLR in recognition of specific molecular structures of lipoproteins, peptidoglycan, dsRNA, LPS and viral RNA. Peptidoglycans from gram-positive bacteria and LPS from gram-negative bacteria bind TLR2 and TLR4, respectively, activate IκB kinases (IKK), and trigger NFκB activation through the MyD88-dependent pathway. The phosphorylation of IκBα by IKK releases it from p50 and p65 of NFκB, allowing for nuclear translocation of NFκB where it binds to promoter regions of many inflammatory genes [90]. Activation of NFκB leads to induction of proinflammatory genes as well as anti-apoptotic genes. An auto-feedback loop exists to downregulate NFκB as IκBα is also induced by NFκB, thereby turning off NFκB activation. TLR4 also signals through TRIF, the adaptor used by TLR3, to induce the expression of IFN-mediated genes [89]. A recent report indicates that TLR2 signaling by cell wall peptidoglycans of *S. aureus* downregulates T cell activation and likely reduces the risk of toxic shock [91].

TNFα binds to TNF receptor 1 (TNFR1), TNFR2 and both receptors use intracellular TRAFs different from those used by IL-1R1 or TLRs but ultimately activating NFκB, resulting in expression of other cytokines, adhesion and co-stimulatory molecules [21,92]. The cytotoxic functions of TNFα are mostly mediated by its binding to TNFR1. Cross-linking receptor chains and clustering upon binding of TNFα results in recruitment of intracellular signaling molecules to the receptor. However, the TNFR possesses death domains, commonly present in receptors of the TNFR superfamily, and binding of TNFα to TNFR1 and TNFR2 also triggers cell death through caspase activation. In this regard, there are common signaling molecules among the TNFR family which includes Fas (CD95), the expression of which is induced by superantigens. Intracellular adaptors, TRADD (TNFR-associated death domain), and FADD (Fas-associated death domain) are used by the TNFR superfamily to activate the caspase 8 cascade, JNK, and NFκB, accounting for the diverse biological effects of TNFα including apoptosis, cell activation, coagulation, inflammation, and host defense [92]. TNFα and IFNγ act synergistically on epithelial cells to increase ion transport, causing cell damage and epithelial leakage [27]. The importance of TNFα in mediating the pathological effects in SEB-induced lethality was recognized early on as anti-TNFα antibodies conferred protection from SEB-induced shock in a D-galactoseamine sensitized mouse model [19]. 

## 7. T Cell Cytokines and Chemokines Activate the PI3K/Akt/mTOR Pathway

SEB binding induces TCR and co-stimulatory molecule CD28 activating PI3K/Akt/mTOR pathway directly by membrane proximal components. In addition, the SEB-induced cytokines IFNγ, IL-2, and chemokines binding to their respective receptors all activate PI3K activity. Diverse stimuli and cytokines initiate the PI3K pathway with some common subsequent steps as well as multiple branch points for regulation of Akt and mTOR, kinases downstream of PI3K.

IFNγ binds to IFNγR, which belongs to the family of interferon receptors, including the structurally different receptors for type 1 interferons [93,94]. IFNRs use very different adaptors and signal transducers from those for IL-1R, or TNFR, with signal transducer and activator of transcription 1 (STAT1) phosphorylation by JAK1 and JAK2 being critical for the IFNγR pathway to activate antiviral responses and expression of other IFNγ-mediated genes. The binding of IFNγ to specific IFNγ R triggers activation of receptor-associated PTK, JAK1 and JAK2. This leads to phosphorylation and activation of STAT1. Dimerization and translocation of STAT1 to the nucleus allows STAT1 to bind and activate IFNγ-specific genes [95]. STAT1 activation is negatively regulated by suppressor of cytokine signaling 1 (SOCS1) and SOCS3. The IFNγ-activated JAKs also activate PI3K in a STAT1 independent manner culminating in mTOR pathway activation, promoting protein translation [95]. IFNγ also activates PKC leading to MAPK pathway activation, which is commonly activated by IL-1, TLR ligands, and TNFα through TRAFs. However, IFNγ induces apoptosis by the induction and activation of death receptors such as Fas, activating FADD and caspase 8 signaling. The activation of caspase 8 cascade results in cytochrome c release from mitochondria and DNA fragmentation. *In vitro*, IFNγ induces MHC class II molecules, immunoproteasome components, and antigen-processing protein transporters to enhance immune responses in host defense [95]. IFNγ dirupts epithelial barrier function and ion transport in superantigen-activated cells and many of the interference of epithelial barrier function *in vitro* can be duplicated with IFNγ with effects synergized by TNFα [96]. Anti-IFNγ inhibited SEB-induced weight loss and hypoglycemia but had no effect on mortality in a D-galactosamine-sensitized mouse model of SEB-mediated shock [97]. 

IL-2 binds to the IL-2R, which consists of three separate chains that heterodimerize and signal through JAK1 and JAK3, activating PI3K and Ras [98]. The activation of the PI3K/Akt/mTOR axis and Ras signaling controls proliferation, growth, and differentiation of many cell types. Ras activates MAPK and ERK cascades leading to activation of AP-1, cJun/Fos and NFAT. IL-2 induces vasodilation and increases microvascular permeability by suppressing endothelin-1, ultimately causing perivascular edema seen in SEB-induced lung injury and shock models [99,100]. A recent study demonstrates the prominent role of IL-2 as IL-2-deficient mice are resistant to SEB-induced toxic shock [101].

IL-6, from both macrophages and activated T cells, has some overlapping activities with IL-1 and TNFα, activates by binding to a different class of receptors belonging to the gp130 family [102]. Binding of IL-6 to its heterodimeric receptor activates JAK3 and Ras. Activated JAK3 phosphorylates STAT3 which then dimerizes and translocates to the nucleus where it binds target genes essential for gp130-mediated cell survival and G1 to S phase transition. The Ras-mediated pathway leads to MAPK activation. In addition, IL-6R also signals through PI3K/Akt/mTOR to promote survival of cells. Together and individually, IL-1, TNFα and IL-6 act on the liver to release acute phase proteins, activate anti-apoptopic pathways, and decrease liver clearance function. 

The chemokines, IL-8, MCP-1, MIP-1α, and MIP-1β, are induced directly by SEB or TSST-1 and selectively act as chemoattractants and activate leukocytes and influence migration of neutrophils, dendritic cells and leukocytes [13,21,103]. Chemokines bind to seven-transmembrane GPCR, induce early Ca++ flux, activate PLC and signal via the PI3K pathway [21,103,104]. Cytokine- and chemokine-activated neutrophils, recruited to sites of tissue injury and inflammation, produce ROS and MMPs contributing to organ dysfunction. MMPs cause tissue degradation and change chemokine interactions with the extracellular matrix creating a local gradient effect of chemokines [103]. Exudates from superantigen-injected air pouches were predominantly neutophils with some macrophages [13]. Endothelial cells surrounding air pouches expressed ICAM-1, TNFα, MIP-2 (an IL-8 related protein in mice), MIP-1α, and JE. Both systemic and intranasal administration of SEB caused acute lung injury characterized by increased expression of adhesion molecules ICAM-1 and VCAM, increased neutrophils and mononuclear cells infiltrate, endothelial cell injury, and increased vascular permeability [18,105].

The PI3K signaling pathway through Akt activation can directly and indirectly modulate mTOR activation. Upstream positive regulators of mTORC1 include PI3K, PDK1, Akt, mTORC2, RHEB, and nutrients leading to increase translation, cell proliferation, and survival. Negative regulators of mTORC1 are AMPK, TSC1/TSC2, and AMP/ATP levels acting in concert to integrate signals controlling cell metabolism, cell survival, and proliferation [80,81]. Since TCR, CD28, IL-2R, IFNγR and chemokine receptors all signal through PI3K/Akt/mTOR, this pathway plays a dominant role in superantigen-induced effects.

## 8. Therapeutic Antibodies against SEB

There is currently no available therapeutics for treatment of superantigen-induced shock except for the use of intravenous human immunoglobulin [106]. Targeting superantigen directly by neutralization of toxins is most suitable at the early stages of exposure before cell activation and release of proinflammatory cytokines. Some of the neutralizing antibodies against one superantigen cross-react and prevent the biological effects of a different superantigen [37]. Various monoclonal and human-mouse chimeric antibodies against SEB have been used effectively to target SEB-induced T cell activation [107,108,109]. A mixture of non-protective monoclonal antibodies was effective in rescuing mice from SEB-mediated shock with one of the antibody inducing a structural change upon binding to SEB which then allowed binding of a different antibody to neutralize SEB [109]. Recombinant mutants of SEB with attenuated binding to MHC class II and devoid of superantigenicity were also used successfully to vaccinate mice and monkeys against SEB-induced disease [110]. *S. aureus* bacteremia triggers antibody response against superantigens and antibody titers increase during infection thereby protecting the host [111]. Carriers previously exposed to *S. aureus* have high titers of neutralizing antibodies specific for the superantigens expressed by their colonizing strain and are protected against *S. aureus* septicemia [112]. 

## 9. Inhibitors of Cell Receptor-Toxin Interaction

Because the binding regions of SEB to MHC class II and TCR are known, small overlapping peptides of SEB can also be used as antagonists to block the initial step of receptor-toxin interactions. Conserved peptides corresponding to residues 150–161 of SEB blocked T cell activation and prevented SEA-, SEB-, or TSST-1-induced lethal shock in mice [113]. This segment of SEB is not associated with the classically defined MHC class II or TCR binding domains, but it may block co-stimulatory signals necessary for T-cell activation. However other investigators found no inhibitory activities with these peptides *in vitro* and *in vivo* [114,115]. Bi-specific chimeric inhibitors composed of the DRα1 domain of MHC class II and Vβdomain of the TCR connected by a flexible GSTAPPA)_2_ linker were reported to bind SEB competitively and prevent its binding to MHC class II of APC and TCR on T cells [116]. Both cell activation and IL-2 production was blocked by the use of these chimeras in SEB-stimulated PBMC. A soluble TCR Vβ mutant with high affinity binding was engineered to neutralize SEB and SPEA [117]. CTLA4-Ig, the synthetic ligand for CD28 inhibited TSST-1-induced T cell proliferation *in vitro* and prevented lethal toxic shock *in vivo* [118]. The recent study of using novel peptides corresponding to the CD28 binding regions to block SEB-mediated effects underscores the importance of co-stimulatory signals in T cell activation by superantigens [52]. Another approach is the use of aptamers, basically peptides or single-stranded nucleic acid, obtained from recombinant libraries, to bind SEB and block interaction with receptor [119]. 

## 10. Inhibitors of Signal Transduction

An important class of therapeutic compounds to be considered is inhibitors that can block signal transduction pathways activated by superantigens, as these events are post-exposure and may be more amenable to suppression and manipulation. The obvious advantage is that they are likely broad spectrum, inhibiting many different superantigens or even pathogens that elicit similar host responses or pathways. *In vitro* studies have shown that many of the genes including cell adhesion molecules, cytokines, chemokines, acute phase proteins, and inducible nitric oxide synthase, implicated in superantigen-induced lethal shock contain NFκB binding sites in the promotor/enhancer region [90]. The activation of NFκB, therefore, leads to the inducible expression of many of the mediators involved in inflammation and tissue injury seen in SEB-induced lung injury and toxic shock models and inhibiting NFκB may be beneficial in preventing superantigen-induced diseases. NFκB binding activity is increased in patients with acute inflammation and sepsis, and can be correlated with clinical severity and mortality [120]. A cell-permeable cyclic peptide targeting NFκB nuclear transport reduced SEB-induced T cell responses and inflammatory cytokine production [121]. Decreased mortality rates accompanied by an attenuation in liver apoptosis and hemorrhagic necrosis were seen in mice given D-galactosamine plus SEB along with this NFκB inhibitor [99]. 

Another potent NFκB inhibitor is dexamethasone, a well-known FDA-approved immunosuppressive corticosteriod used clinically to treat various inflammatory diseases [122]. Dexamethasone potently inhibited SEA-, and SEB-induced cytokine release, T-cell proliferation, and cell activation marker expression in human PBMC [123]. Dexamethasone also significantly reduced serum levels of TNFα, IFNγ, IL-1, IL-2, and IL-6 in the LPS-potentiated SEB model and the un-potentiated SEB-only model of toxic shock [105,124]. Importantly, dexamethasone decreased mortality in both of these mouse models was accompanied by attenuation of the hypothermic response and weight loss induced by SEB. Another NFκB and proteosome inhibitor, bortezomib, attenuated SEB-induced cytokine release but had no effect on SEB-induced lethality and liver necrosis [125]. Polyphenols such as epigallocatechin gallate (EGCG) from green tea and resveratrol (RES) from red wine also reduced superantigen-induced T cell proliferation and cytokine release from human PBMC by decreasing NFκB activity [126]. EGCG suppressed T cell activation, reducing IFNγ and TNFα from SEB-stimulated human PBMC and murine lymph node cells and reduced IFNγ-induced epithelial permeability increases [127]. RES blocked SEB-induced T cell activation, pulmonary permeability increases, caspase 8-dependent apoptosis, and prevented SEB-induced lung injury in mice [128]. Recently, a synthetic mimetic (EM-163) to the BB-loop of MyD88 was found to inhibit TNFα, IFNγ, IL-1, IL-2 and IL-6 in human PBMC activated by superantigens [129]. Furthermore, EM163 reduced the level of cytokines in serum and protected mice from LPS plus SEB-induced shock [129,130]. 

Other signal transduction inhibitors include those directed against PKC and PTK. H7, a PKC inhibitor and genistein, a tyrosine kinase inhibitor each blocked TNFα but not IL-1 production from TSST-1-stimulated PBMC [131]. D609, an inhibitor of PLC, which is activated upon superantigen binding to TCR and CD28, blocked SEB-induced effects both *in vitro* and *in vivo* [132,133]. Curiously, the serum level of TNFα in mice treated with D609 and superantigen remained high despite reduction in lethality [133]. Another natural feedback inhibitor of the various STATs used by IFNγ and IL-2 signaling is SOCS3 which therefore controls the effects of these two cytokines [93]. In this regard, a cell-penetrating form of SOCS3 protected animals from lethal effects of SEB and LPS by reducing production of inflammatory cytokines and attenuating liver apoptosis and hemorrhagic necrosis [134]. 

Two other potent immunosuppressant and calcineurin inhibitor used clinically for preventing transplant rejection, cyclosporine A (CsA) and tacrolimus, did not protect superantigen-induced shock in monkeys and human HLADR3-transgenic mice, respectively [135,136]. CsA inhibited SEB-induced T cell proliferation *in vitro* and reduced serum IL-2, TNFα, and IFNγ, as well as attenuated pulmonary inflammation which did not translated to a protective effect [135]. Tacrolimus suppressed superantigen-induced T cell activation *in vitro* but did not confer protection from shock *in vivo* [136]. 

Recently, the mTORC1 specific inhibitor, rapamycin was shown to be efficacious even when given 24 h after SEB in a murine model of SEB-induced shock [87]. Rapamycin is a FDA-approved drug currently used to prevent kidney graft rejection and is under clinically trials for cancer treatment. Rapamycin works by suppressing mTOR activities resulting in inhibition of SEB-induced T cell proliferation, reduced IL-2 and IFNγ. Another study indicates rapamycin was effective as an intranasal drug, providing practical protection against SEB-induced shock even 17 h after toxin exposure [137].

Oxidative stress is another hallmark of SEB-intoxication as systemic administration of SEB causes prolonged lung inflammation that is difficult to resolve [105]. Acute lung injury arises as SEB-, cytokine- and chemokine-activated neutrophils infiltrate into lung areas, produce high levels of ROS which in turn cause increase in vascular permeability and apoptosis [18]. One strategy is the use of anti-oxidants such as *N*-acetyl cysteine (NAC) and pyrrolidine dithiocarbamate (PDTC) to mitigate oxidative stress. Both NAC and PDTC are FDA-approved drugs for other indications and prevented NF-κB signaling in superantigen-activated human PBMC [138]. 

Dexamethasone, rapamycin, cyclosporine A, tacrolimus, bortezomib, NAC, PDTC are FDA-approved drugs currently used for other indications. The testing of FDA-approved drugs for preventing superantigen-induced shock should speed up the approval process for biodefense use in case of exposure. However, as seen from the various examples above, even knowing the mechanism of action of these drugs is no guarantee for success as *in vivo* dosages, dosing routes and schedules as well as animal models all affect the outcome. Rapamycin, by decreasing the levels and effects of IL-2 and IFNγ through mTOR inhibition, is proven to be effective to counter the toxic effects of SEB.

## 11. Inhibitors of Cytokines

Due to the pathophysiological complexities of toxic shock resulting from excessive proinflammatory cytokine release from host cells responding to superantigens, therapeutics aimed at inhibiting the release of these mediators overlap with inhibitors of signal transduction pathways used by these cytokines. Most therapeutic testing in animal models of SEB-induced shock have targeted proinflammatory cytokines, as there is a strong correlation between toxicity and increased serum levels of these inflammatory mediators [12,13,14,15,16]. Neutralizing antibodies against TNFα prevented SEB-induced lethality in D-galactoseamine sensitized mouse model establishing the critical role of TNFα in lethal shock [19]. The anti-inflammatory cytokine IL-10 also reduced lethality to superantigen-induced toxic shock by reducing the production of IL-1, TNFα and IFNγ [139,140]. Niacinamide, a nitric oxide inhibitor, mitigated the effects of SEB by inhibiting the production of IL-2 and IFNγ, and improved survival of mice given LPS plus SEB [141]. Other drugs tested to block cytokine release from superantigen-activated cells include doxycycline, an antibiotic, and pentoxyfylline, a methylxanthine derivative. Doxycycline blocked SEB-induced proinflammatory cytokines and chemokines and T-cell proliferation in human PBMC [142]. Pentoxyfylline, a phophodiesterase inhibitor, is used clinically to treat peripheral vascular disease as it disrupts intracellular regulatory pathways that affect leukocyte adhesion and cytokine production. Pentoxyfylline reduced cytokines and T cell proliferation in SEB- or TSST-1-stimulated cells. It prevented lethal shock accompanied by reduction in serum cytokines in the LPS plus SEB mouse model [143].

Another strategy to attenuate IL-1 release from superantigen-activated cells is to target caspase 1, a proteolytic enzyme that cleaves pro-IL-1 into active IL-1 [21]. The caspase 1 specific inhibitor, Ac-YVAD-cmk, attenuated both IL-1 and MCP production in SEB- and TSST-1 stimulated PBMC cultures but had no effect on other cytokines or T-cell proliferation [144]. Caspase 3 and caspase 8 inhibitors were also ineffective in down-regulating superantigen-activated cells or T cell proliferation. In contrast, a pan-caspase inhibitor, Z-D-CH_2_-DCB, reduced the production of IL-1β, TNFα, IL-6, IFNγ, MCP, MIP-1α, MIP-1β, and inhibited T-cell proliferation in SEB- and TSST-1-stimulated PBMC [144]. 

Other compounds tested against superantigen-induced effects include herbal medicinal compounds, tryptanthrin and baicalin. Tryptanthrin, derived from an Asian medicinal plant, *Isatis tinctoria,* reduced IFNγ production by SEB-stimulated lymphocytes from Peyer’s patches [145]. Baicalin, a flavone isolated from the Chinese medicinal herb *Scutellaria baicalensis*, attenuated IL-1, TNF, IL-6, IFNγ, MCP-1, MIP-1α, MIP-1β mRNA and protein expression in SEB- and TSST-1-stimulated human PBMC and blocked T cell proliferation [146]. Herbal compounds are usually less-well characterized and are often used in combination with other medicinal herbs to be effective. 

## 12. Summary

By binding to both MHC class II and TCR, superantigens stimulate T-cell proliferation and excessive release of multiple inflammatory cytokines and chemokines. Similar to other shock syndromes, extensive tissue inflammation and injury is the result of superantigen-induced proinflammatory mediators via NFκB activation. The three signals for T cell activation after superantigen binding all activate the PI3K/Akt/mTOR pathway by sequential phosphorylation steps regulating proliferation, growth and survival. T cell cytokines, IFNγ and IL-2 and chemokines signal via the PI3K/Akt/mTOR pathway, making this pathway even more important as a target for intervention. The ability to stop the inflammatory and proliferation/survival signals initiated by superantigens appears to be critical in preventing superantigen-mediated shock.
